# Low-Intensity and Chemo-Free Treatments in Ph+ ALL: Progression-Free Survival Based on Indirect Comparisons

**DOI:** 10.3390/hematolrep15040068

**Published:** 2023-11-26

**Authors:** Melania Rivano, Daniele Mengato, Marco Chiumente, Andrea Messori

**Affiliations:** 1 Binaghi Hospital, 09126 Cagliari, Italy; melania.rivano@gmail.com; 2 Hospital Pharmacy Department, Azienda Ospedale—Università of Padova, Via Giustiniani 2, 35128 Padua, Italy; daniele.mengato@gmail.com; 3 Scientific Direction, Società Italiana di Farmacia Clinica e Terapia (SIFaCT), 10123 Torino, Italy; marco.chiumente@sifact.it; 4 HTA Unit, Regional Health Care System, Regione Toscana, 50139 Firenze, Italy

**Keywords:** acute lymphoblastic leukemia, imatinib, dasatinib: nilotinib, ponatinib, blinatumomab, reduced-intensity chemotherapy, chemotherapy-free, progression-free

## Abstract

In Philadelphia chromosome-positive B-cell (Ph+) acute lymphoblastic leukemia (LLA), growing evidence has accumulated regarding the efficacy of low-intensity and chemo-free regimens. Our objective was to analyze all recent trials evaluating these treatments and to compare them in terms of efficacy. We applied the Shiny method, an artificial intelligence technique, to analyze Kaplan–Meier curves and reconstruct patient-level data. Reconstructed patient data were then evaluated through standard survival statistics and subjected to indirect head-to-head treatment comparisons. The endpoint was progression-free survival (PFS). Based on 432 reconstructed patients, eight trials were analyzed. The survival data from these trials were pooled into three types of treatments: (i) treatments based on tyrosine kinase inhibitors (TKIs) combined with reduced-intensity chemotherapy (denoted as TKICHE); (ii) TKIs associated with steroids with no chemotherapy (TKISTE); (iii) chemotherapy-free combinations of blinatumomab plus TKIs (TKIBLI). According to the Shiny method, the three PFS curves were reported in a single Kaplan–Meier graph and subjected to survival statistics. In terms of PFS, TKIBLI ranked first, TKICHE second, and TKISTE third; the differences between these three regimens were statistically significant. This multi-treatment Kaplan–Meier graph, generated through the Shiny method, summarized the current evidence on these treatments in both qualitative and quantitative terms.

## 1. Introduction

The Philadelphia (Ph) chromosome represents the most frequent cytogenetic abnormality in adults with acute lymphoblastic leukemia (ALL), with an incidence that increases with age, reaching approximately 50% in patients aged 60 years and older [1,2,3]. The combination of the Ph chromosome and BCR-ABL fusion gene is associated with the most unfavorable outcome, irrespective of age [4].

Induction chemotherapy rarely determines a sustained complete remission in these patients, and so, after complete hematologic remission has been achieved, an allogeneic stem cell transplant, when feasible, represents the only possibility of cure [5]. Treatment options for Ph+ ALL have expanded over the past 15 years, mainly due to the advent of tyrosine kinase inhibitors (TKIs) that have significantly improved outcomes in various combination regimens [6,7,8].

The first-generation TKI (imatinib), the second-generation TKIs (dasatinib and nilotinib), and the third-generation TKI (ponatinib) have successfully been combined with chemotherapy in prospective studies conducted in adult patients with Ph+ ALL [9,10,11]. More recently, a further improvement was achieved with a chemo-free induction/consolidation strategy based on the combination of a TKI with the CD3/CD19 bispecific antibody blinatumomab [12,13]. Overall, these novel regimens have determined very substantial improvements in the progression-free survival (PFS) of these patients.

In the field of survival analysis, important methodological improvements have occurred in the past two years. In particular, the IPDfromKM method [14] (also known as “Shiny method”) has established itself as a powerful tool to reconstruct individual patient data from the graphs of Kaplan–Meier curves. The main characteristic of the Shiny method is that each Kaplan–Meier curve is analyzed through artificial intelligence software that reconstructs patient-level data over the entire study follow-up. In this way, treatments can be compared indirectly with each other, and the results can be interpreted by the application of standard survival statistics. Despite its theoretical complexity, the Shiny method is extremely easy to use. In fact, only three pieces of information are needed to generate a patient database from a Kaplan–Meier curve: (i) the graph of the curve; (ii) the total number of patients for the curve concerned; (iii) the total number of events. A wide range of experience has rapidly accumulated in the use of this approach, particularly in the area of anti-cancer agents [15]. In this report, we applied the Shiny method to analyze the most recent survival studies focused on the treatment of Ph+ ALL with TKIs combined with low-intensity regimens, steroids, and chemo-free approaches.

## 2. Materials and Methods

### 2.1. Study Design

Our analysis aimed to retrieve updated information on novel therapeutic approaches for ALL and study, in comparative terms, the survival outcomes observed with these treatments. After a standard PubMed search, the datasets suitable for our analysis were identified. Our analysis included the datasets in which the information on PFS was reported (with follow-up of at least 2 years), and the graph of the Kaplan–Meier curve was available. As proposed in a recent review by Haddad et al. [9], these datasets were grouped as follows: (i) treatments based on TKIs combined with reduced-intensity chemotherapy (denoted as TKICHE); (ii) TKIs associated with steroids with no chemotherapy (denoted as TKISTE); (iii) chemotherapy-free combinations of blinatumomab plus TKIs (denoted as TKIBLI). The trials evaluating these three types of treatment were subjected to the procedure of individual-patient data reconstruction according to the Shiny method. Thereafter, the treatments identified as TKICHE, TKISTE, and TKIBLI were compared with each other using standard survival statistics. Our endpoint was PFS. The results of our analysis were summarized in a multi-trial Kaplan–Meier graph generated on the basis of all reconstructed patients. 

### 2.2. Literature Search

**Figure 1:**
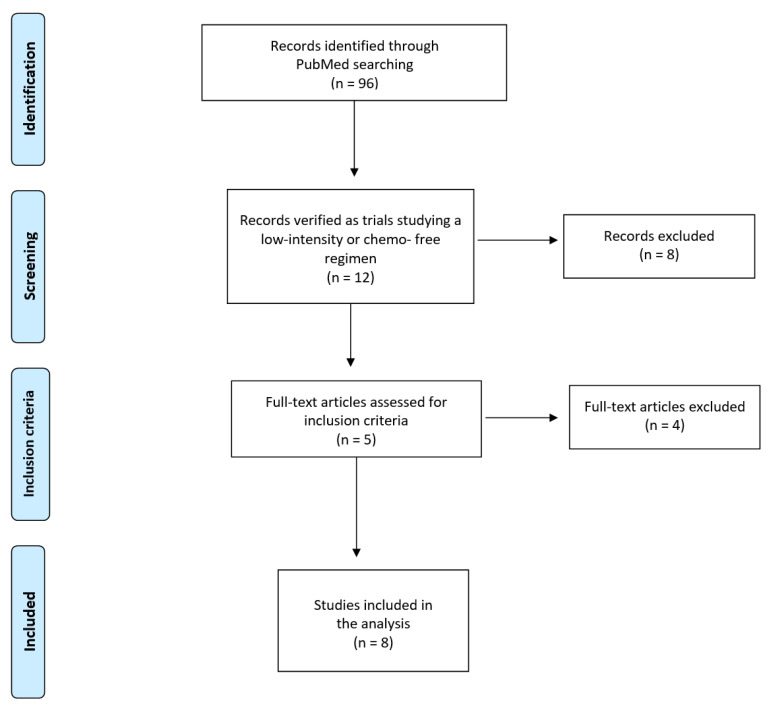
Literature search: flow of trial selection based on the PRISMA algorithm. See also the Supplementary Material.

Our PubMed search covered the time interval from PubMed inception to October 2023 (date of the last search: 29 October 2023; keywords: (leukemia OR leukaemia) AND “progression-free” AND (imatinib OR dasatinib OR nilotinib OR ponatinib); filter: “clinical trial”). Original clinical trials were eligible for further scrutiny. A further selection of the literature identified some recent trials cited in the recent review published by Haddad et al. [9]. When duplicate citations were found for the same trial, only the most updated dataset was included in our analysis. The trial selection was performed according to the Preferred Reporting Items for Systemic Review and Meta-Analyses (PRISMA) approach [16]. Figure 1 shows the flow of trial selection based on the PRISMA algorithm.

### 2.3. Reconstruction of Individual Patient Data from Kaplan–Meier Survival Curves

We used the Shiny method [14], which was implemented as in the other analyses published by our research group [15]. Firstly, patient-level data were reconstructed from each of the treatment arms of the original trials. Thereafter, in cases where similar or identical treatments were investigated in different trials, the reconstructed patients were pooled into a single patient group. In this way, three treatment groups were formed for TKICHE, TKISTE, and TKIBLI. Finally, these three treatment groups were subjected to standard survival statistics, in which PFS was the endpoint.

### 2.4. Survival Statistics

Survival statistics were carried out by standard methods using the Cox model. Head-to-head comparisons were assessed according to the hazard ratio (HR) with 95% confidence interval (CI). Statistical analyses based on reconstructed patient-level data were conducted under the R-platform as in our previous analyses [15].

### 2.5. Assessment of Heterogeneity in Studies Pooled Together

In three separate analyses, we examined the degree of heterogeneity within the trials assigned to the TKICHE, TKISTE, and TKIBLI groups, respectively. Heterogeneity was likely to depend mainly on differences in patients’ inclusion criteria. Furthermore, in each of these three groups, heterogeneity was assessed through a post hoc analysis aimed at estimating the degree of concordance between similar studies expected to report similar survival patterns. For this purpose, the likelihood ratio test and the concordance test were employed. Further details about this assessment of heterogeneity are presented in Appendix A.

### 2.6. Data Sharing Statement 

For each treatment under comparison, the database of reconstructed patients is available from the author upon request.

## 3. Results

Our PubMed search identified 96 studies (Figure 1). Of these, 12 trials [17,18,19,20,21,22,23,24,25,26,27,28] were eligible for our analysis because they investigated a treatment based on a low-intensity or a chemo-free regimen. Among these 12 studies, the trial by Ottman et al. [17] was excluded owing to the absence of the Kaplan–Meier curve; likewise, the study by Short et al. [28] was excluded owing to the absence of information on PFS. The study by Chalandon et al. [18] was excluded because only induction was not intensive. Hence, a total of eight studies and 432 patients (Table 1) were selected for our analysis [19,20,21,22,23,24,25,26]. 

The following agents were investigated in these eight trials:

Trial (a): dasatinib in combination with low-intensity chemotherapy (Rousselot et al. [19]);

Trial (b): nilotinib combined with low-intensity chemotherapy (Rousselot et al. [20]);

Trial (c): dasatinib plus steroids induction followed by dasatinib alone (Chiaretti et al. [21]);

Trial (d): imatinib combined with steroids (Vignetti et al. [22]);

Trial (e): dasatinib induction therapy combined with steroids (Foà et al. [23]);

Trial (f): ponatinib plus prednisone (Martinelli et al. [24]);

Trial (g): dasatinib plus blinatumomab (Chiaretti et al. [25]);

Trial (h): ponatinib plus blinatumomab (Jabbour et al. [26]).

According to our study protocol, individual patient data from these eight trials were reconstructed by application of the Shiny method. Then, the treatments from different trials belonging to the same pharmacological class were pooled into a single patient group. The following three groups were formed:

(1) The regimen denoted TKICHE (i.e., TKI plus low-intensity chemotherapy), which includes four trials, namely dasatinib plus low-intensity chemotherapy in the trial by Rousselot et al. [19], nilotinib plus low-intensity chemotherapy in the GRAAPH-2014 Study by Rousselot et al. [20], and dasatinib plus steroids induction in the trial by Chiaretti et al. [21].
(2) The regimen denoted TKISTE (i.e., TKIs combined with steroids), which includes three trials, namely those by Vignetti et al. [22], Foà et al. [23], and Martinelli et al. [24].
(3) The regimen denoted TKIBLI (i.e., TKI plus blinatumomab), which includes two trials, namely blinatumomab combined with the second-generation dasatinib, as reported by Chiaretti et al. [25], and blinatumomab combined with the third-generation ponatinib, as reported by Jabbour et al. [26].

The assignment to TKICHE of the trial by Chiaretti et al. [21] can be a matter of controversy because the regimen of this trial was chemo-free rather than based on low intensity. Consistently with the review by Haddad et al. [9], we kept this trial by Chiaretti in the TKICHE group.

**Figure 2:**
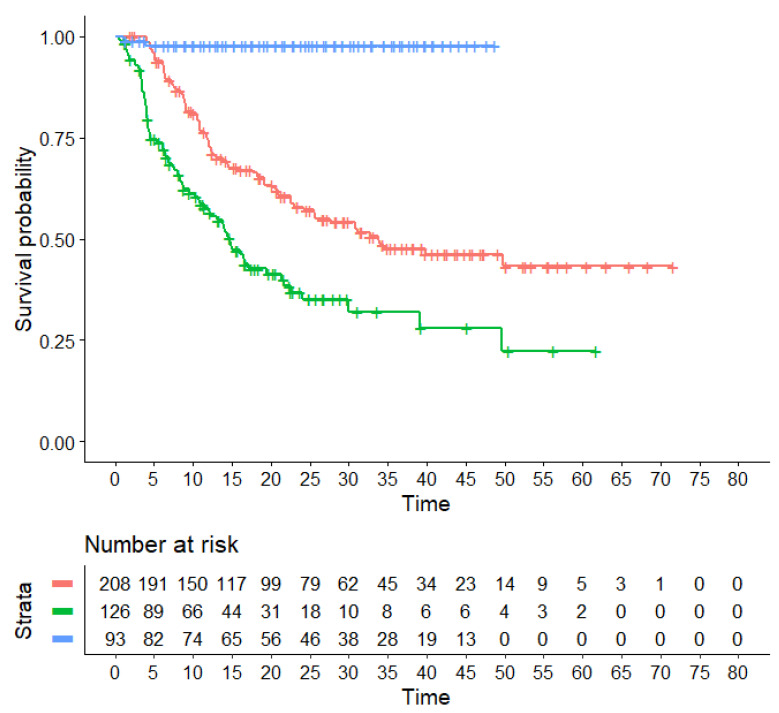
Kaplan–Meier survival curves generated from reconstructed patients for three combination regimens. Treatments from different trials belonging to the same pharmacological class were pooled into a single patient group (TKICHE, TKISTE, or TKIBLI). In red, TKI plus chemotherapy (TKICHE); in green, TKI plus steroids (TKISTE); in blue, TKI plus blinatumomab (TKIBLI). Time in months, endpoint PFS.

In our main analysis, these three regimens (TKICHE, TKISTE, and TKIBLI) were compared with one another based on the endpoint of PFS. The results of this analysis are shown in Figure 2, which presents the Kaplan–Meier curves generated for these three regimens from reconstructed patients. In the statistical comparisons across these three regimens, the following values of HR were estimated:

- HR for the comparison of TKICHE vs. TKISTE: 0.5066 (95% CI, 0.3705 to 0.6927; *p* = 0.00002);
- HR for the comparison of TKIBLI vs. TKICHE: 0.448 (95% CI, 0.0110 to 0.182; *p* = 0.00014);
- HR for the comparison of TKIBLI vs. TKISTE = 0.023 (95% CI, 0.0053 to 0.096; *p* < 0.001).

All these three values of HR are statistically significant. Medians for these three regimens were 33.7 months for TKICHE (95% CI, 25.5 to not computable), 14.7 months for TKISTE (95% CI, 11.5 to 21.7), and not computable for TKIBLI. According to these results, the advantage in PFS for TKIBLI compared with the other two regimens has a remarkable clinical relevance, along with its high level of statistical significance. 

Finally, besides our main analysis, we separately assessed the degree of heterogeneity for the three regimes (TKICHE, TKISTE, and TKIBLI). For this purpose, we carried out three post hoc analyses in which the likelihood ratio test was estimated. These post hoc analyses are presented in detail in Appendix A. As expected, in the overall analysis of these eight trials, the between-trial heterogeneity was highly significant (concordance = 0.687 with standard error = 0.019; likelihood ratio test = 97.96 on 2 df, *p* < 0.001; Wald test = 40.92 on 2 df, *p* < 0.001).

## 4. Discussion

Elderly or unfit patients are not candidates for intensive chemotherapy owing to the high risk of morbidity and mortality. Hence, lower-intensity regimens have been designed especially for these patients [19], even though they have also been explored in younger, more fit patients with newly diagnosed Ph+ ALL [21]. Similarly, regimens based on induction therapy with steroids plus TKIs have mainly been tested in elderly or unfit patients [22,23,24].

Findings from these studies suggest that low-intensity therapies are safe and feasible in patients with Ph+, particularly those who are older and/or unfit for intensive chemotherapy or allogeneic stem cell transplantation (allo-SCT). On the other hand, blinatumomab has initially been shown to be highly effective as a single agent in patients with relapsed/refractory Ph+ ALL [27]; thereafter, blinatumomab was investigated in combination with a TKI [25,26], determining excellent results. The two trials by Chiaretti et al. [25] and by Jabbour et al. [26] are those included in our TKIBLI regimen. 

Our analysis based on 432 patients reconstructed from eight trials showed that the chemo-free induction/consolidation strategy, based on the sequential administration of dasatinib or ponatinib followed by blinatumomab (i.e., the TKIBLI regimen), demonstrates a clear superiority compared with TKICHE or TKISTE regimens. For example, in the updated analysis of the GIMEMA LAL2116 trial [13], Chiaretti et al. [25] reported very favorable outcomes, with an estimated overall survival of 78% (95% CI, 66–92%) at 48 months and DFS of 75% (95% CI, 64–87%). While these findings are impressive, one should keep in mind the limited size of the patient groups enrolled in the two trials by Chiaretti et al. [25] and Jabbour et al. [26]. 

Also, the results presented at the 2022 European Hematology Association (EHA) Congress by Short et al. [28] confirm that a chemotherapy-free regimen of simultaneous ponatinib and blinatumomab is safe and effective for newly diagnosed Ph+ ALL; the 2-year event-free survival (EFS) and overall survival were both 93% while in the relapsed/refractory Ph+ ALL cohort, the 2-year EFS rate was 42%, and the 2-year overall survival rate was 61%. 

Our analysis has some limitations. The first limitation is the presence of different eligibility criteria among the trials that we pooled into the same group. For example, regarding the TKICHE group of trials, patients older than age 55 years were eligible in the study by Rousselot et al. [19]. In the Graaph-2014 study, Rousselot et al. [20] included patients aged 18 to 60 years. 

Furthermore, regarding the TKIBLI group, patients had a median age of 54 years (range, 24–82) in the GIMEMA LAL2217 trial [25], while in the study by Jabbour et al. [26], the patients had a median age of 51 years and required to have a performance status of ≤2 without comorbidities. These characteristics of the patients included in the TKIBLI trials may have influenced the favorable outcome of this therapeutic approach compared with TKICHE and TKISTE. Finally, some caution is warranted in interpreting the results of the two TKIBLI trials because the overall number of enrolled patients was limited.

Another aspect that deserves specific comments is safety. Chemotherapy-free regimens with blinatumomab combined with second- or third-generation TKIs permit avoiding cytotoxic therapies, thus contributing to improved prognosis. While most imatinib adverse events tend to be mild and often resolve spontaneously, rare but serious side effects have occasionally been reported with later-generation TKIs [29]. Since most patients who do not undergo allo-SCT are recommended to receive indefinite TKI therapy, significant open questions remain concerning long-term outcomes in ALL patients besides mortality. Further prospective studies are needed to identify patients who can safely discontinue TKIs, an option that gains increasing interest with time.

The exclusion of 84 studies selected by our initial PubMed search (see the PRISMA flowchart in Figure 1) deserves some comments. While this number of excluded studies is high, all of these studies were based on patients whose characteristics differed to a great extent from those enrolled in the eight included studies. From this point of view, the main limitation is that while the standard of care for these patients is high-dose therapy followed by HSCT, this treatment was not administered in any of the eight included studies. Hence, this limitation about the choice of the treatments in the 8 included studies was probably more relevant than the limitations affecting the 84 excluded studies in terms of the characteristics of the patients. 

Furthermore, while the standard of care for these patients is recognized as high-dose therapy followed by allo-HSCT, one limitation is that this standard of care was not included in our survival analysis. On the other hand, a survival analysis based on the above-mentioned standard of care is not feasible because the included study had not investigated any patients treated this way. Likewise, converting our article into a systematic review has not been possible considering the 10-day deadline set by the Editor-in-Chief, but, of course, we will be glad to make this change (e.g., by introducing a section about the quality of evidence for the eight trials), if this type of revision is considered mandatory and an adequate deadline for our resubmission is granted.

Another important limitation of our study is that there was no way to remove allogeneic stem cell transplant as a confounder of our statistics. Unfortunately, this drawback could not be corrected because it was not possible to determine who proceeded to undergo allo-SCT on an individual patient basis. While this certainly influenced the outcome of survival, the impact of this confounder would have been more relevant using overall survival as the endpoint of the survival statistics; by contrast, the endpoint of our analysis was progression-free survival.

## 5. Conclusions

In conclusion, major progress has been achieved in the management of Ph+ ALL. After the combination of TKIs with low-intensive chemotherapy or steroids has considerably improved long-term survival, chemotherapy-free regimens with blinatumomab and TKIs seem to represent a further advancement that may revolutionize the landscape of Ph+ ALL. In particular, the combination of second-generation dasatinib or third-generation ponatinib plus blinatumomab is associated with deep and durable remissions while avoiding cytotoxic therapies and mitigating the need for allo-SCT. Our results, obtained from reconstructed patient data, suggest that this strategy might be the most effective; on the other hand, the regimens based on blinatumomab still require further investigation because this option is presently supported only by small-size studies. Finally, the present analysis confirms the good performance of the Shiny method in improving the analysis and the interpretation of survival results in hematologic malignancies [30,31,32,33].

## Figures and Tables

**Table 1:** Main characteristics of the 8 included trials.

	First Author	Year	Trial	Treatment	Inclusion Criteria	Events	No of Patients	Notes
TKICHE	Chaladon et al. [18]	2015	GRAAPH-2005 study	Imatinib combined with low-intensity chemotherapy	Patients aged 18 to 59 years with newly diagnosed Ph1 and/or BCR-ABL1-positive ALL were eligible	65	135	Excluded from our analysis because only induction was not intensive
	Rousselot et al. [19]	2016	EWALL-PH-01 international study	Dasatinib in combination with low-intensity chemotherapy	Patients aged 55 years or older were eligible if they had newly diagnosed Ph1 and/or BCR-ABL ALL	40	71	Included
	Rousselot et al. [20]	2021	Graaph-2014 Study	Nilotinib combined with low-intensity chemotherapy	Ph-positive ALL patients aged 18–60 years old were randomized	23	79	Included
	Chiaretti et al. [21]	2020	GIMEMA LAL1509	Dasatinib plus steroids induction followed by dasatinib alone	Adult Ph+ ALL patients (18–60 years).	13	58	Included
TKISTE	Vignetti et al. [22]	2007	GIMEMA LAL0201-B	Imatinib combined with steroids	Patients with a diagnosis of ALL who were older than 60 years were eligible if they carried either the Ph chromosome or the BCR-ABL molecular translocation	16	29	Included
	Foà et al. [23]	2011	GIMEMA LAL1205	Dasatinib induction therapy combined with steroids	Patients 18 years of age or older (with no upper age limit) were eligible if they had been diagnosed with Ph/BCR-ABLALL	23	53	Included
	Martinelli et al. [24]	2022	GIMEMA LAL 1811	Ponatinib plus prednisone	Patients had new-onset Ph+ ALL and were ≥60 years or were ≥18 years old but unfit for a program of intensive chemotherapy and SCT	34	44	Included
TKIBLI	Chiaretti et al. [25]	2022	GIMEMA LAL2217	Dasatinib + blinatumomab	Ph-positive ALL patients, median age was 54 years (24–82; no upper age limit)	9	58	Included
	Jabbour et al. [26]	2023	NCT03263572	Ponatinib + blinatumomab	Patients with newly diagnosed, relapsed/refractory Ph+ ALL or CML in lymphoid blast phase	2	40	Included

**Figure A1:**
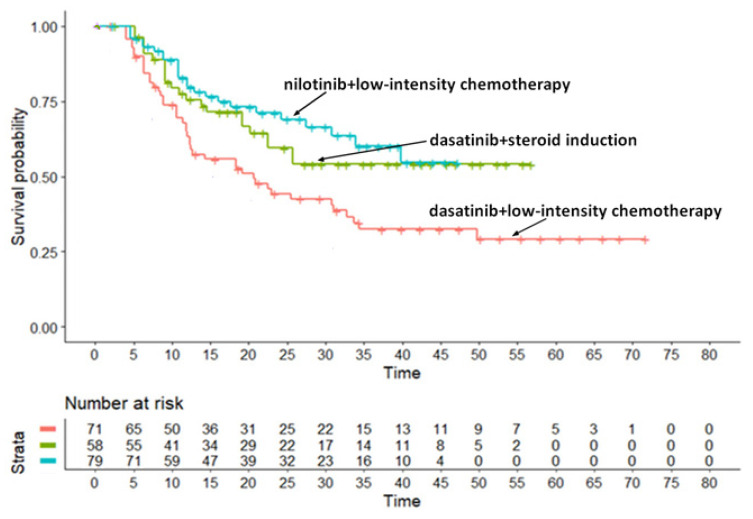
Post hoc analysis for the TKICHE regimen. The treatments shown in this Kaplan–Meier graph include dasatinib in combination with low-intensity chemotherapy (Rousselot et al. [19], Trial (a)) in red, dasatinib plus steroids induction followed by dasatinib alone (Chiaretti et al. [21], Trial (c)) in blue, and nilotinib combined with chemotherapy (Rousselot et al. [20], Trial (b)) in green. Trials (b) and (c) were not significantly different, whereas Trial (a) differed significantly from both Trials (b) and (c). Time in months, endpoint PFS.

**Figure A2:**
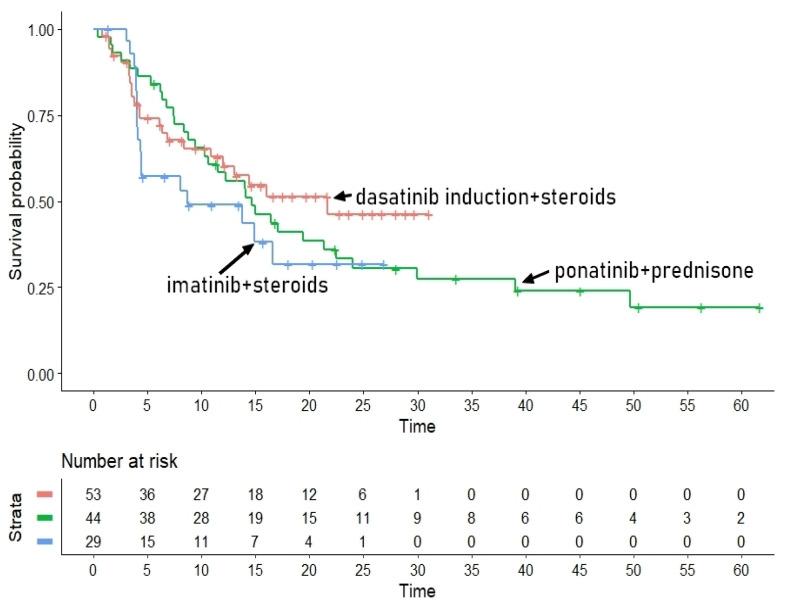
Post hoc analysis for the TKISTE regimen. In blue, imatinib combined with steroids (Vignetti et al. [23], Trial (f)); in red, dasatinib induction therapy combined with steroids (Foà et al. [24], Trial (d)); in green, ponatinib plus prednisone (Martinelli et al. [25], Trial (e)). Time in months, endpoint DFS.

**Figure A3:**
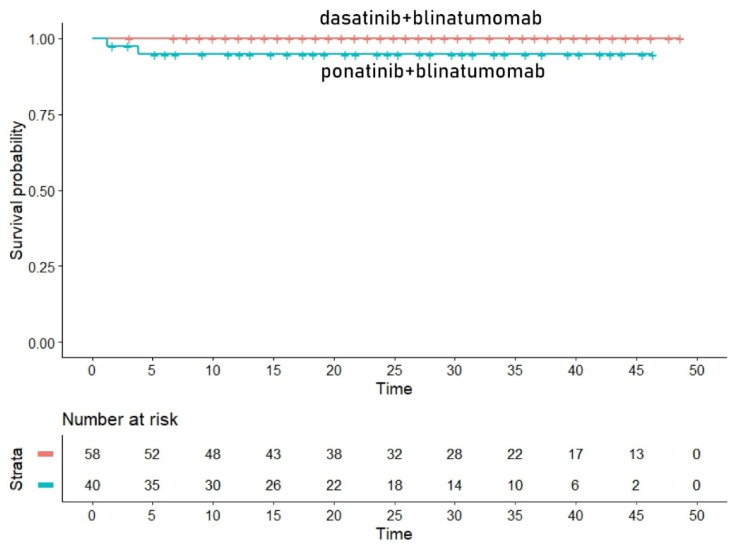
Post hoc analysis for the TKIBLI regimen. In red dasatinib plus blinatumomab (Chiaretti et al. [25], trial (h)) and in blue ponatinib plus blinatumomab (Jabbour et al. [26], trial (i)). Time in months, endpoint DFS.

## Data Availability

For each of the nine treatments under comparison, the database of reconstructed patients is available from the authors upon request.

## Supplementary Materials

The following supporting information can be downloaded at: https://www.mdpi.com/article/10.3390/hematolrep15040068/s1, File S1: PRISMA 2020 Checklist [34].

## Appendix A

### Appendix A.1. Indirect Head-to-Head Efficacy Comparisons for the Three Regimens (TKICHE vs. TKISTE vs. TKIBLI)

This Appendix describes the methods and the results of the post hoc analyses carried out for each of the three combination treatment regimens (TKICHE, TKISTE, and TKIBLI). PFS was the endpoint.

In these three post hoc analyses, the regimens studied in at least two different trials were pooled into a single patient group provided that the treatments belonged to the same pharmacological class, i.e., TKICHE, TKISTE, or TKIBLI. This choice to pool similar (or even identical) combination treatments from different trials was aimed at avoiding an excessive fragmentation of our statistical results but exposed our analysis to an increased risk of underestimating between-trial variability. To manage this issue, each case of data pooling of similar or identical treatments across at least two trials was investigated by a further post hoc PFS analysis, in which the survival data from the different trials were kept separate, and heterogeneity was assessed formally. In these post hoc analyses, the patient inclusion criteria of individual trials were also reviewed; this information has already been reported in narrative form in Table 1. Heterogeneity in these three analyses was assessed according to the likelihood ratio and concordance test.

### Appendix A.2. Survival Analysis for the TKICHE Regimen (Three Trials)

The first group of similar/identical treatments included the combination of TKIs plus chemotherapy according to four trials, namely dasatinib + chemotherapy in the EWALL-PH-01 trial by Rousselot et al. [19], nilotinib + chemotherapy in the Graaph-2014 Study by Rousselot et al. [20], and dasatinib+steroid induction followed by dasatinib in the LAL1509 trial by Chiaretti et al. [21]. The post hoc analysis for these regimens is described in Figure A1. Medians of PFS for these four patient groups were the following:

- Trial (a): 20.7 months (95% CI, 12.3 to 33.7 months) [19];
- Trial (b): not computable (95% CI, 22.5 months to not computable) [20];
- Trial (c): not computable (95% CI, 34.0 months to not computable) [21].

Indirect head-to-head comparisons gave the following values of HR:

- Trial (b) vs. Trial (a): HR, 0.5693 (95% CI, 0.3411 to 0.9501; *p* = 0.031);
- Trial (c) vs. Trial (a): HR, 0.470 (95% CI, 0.2856 to 0.7746; *p* = 0.003);
- Trial (c) vs. Trial (b): HR, 0.826 (95% CI, 0.404 to 1.688; *p* > 0.05).

As shown above, among the three head-to-head indirect comparisons, two were significant, whereas the third (comparison of (c) vs. (b)) was not. The likelihood ratio test showed quite strong heterogeneity (likelihood ratio test = 10.14 on 2 df, *p* < 0.001), which likely depends on the presence of the two significantly different comparisons. Concordance was 0.594 (standard error = 0.03).

### Appendix A.3. Survival Analysis for the TKISTE Regimen (Three Trials)

The second group of pharmacologically similar treatments included the combination of TKIs plus steroids, for which three trials were available, namely those by Vignetti et al. [22], Foà et al. [23], and Martinelli et al. [24]. The post hoc analysis for this regimen is described in Figure A2.

Medians of PFS for these three patient groups were the following:

- Trial (d): 21.67 months (95% CI, 11.97 to not computable);
- Trial (e): 14.69 months (95% CI, 10.65 to 24.0);
- Trial (f): 8.66 months (95% CI, 4.38 to not computable).

The values of HR for the indirect head-to-head comparisons were the following:

- Trial (f) vs. Trial (d): HR= 1.22 (95% CI, 0.71 to 2.11);
- Trial (e) vs. Trial (d): HR= 1.53 (95% CI, 0.82 to 2.88);
- Trial (f) vs. Trial (e): HR = 1.26 (95% CI, 0.55 to 2.89).

Despite the different medians of the three trials, the three head-to-head indirect comparisons did not achieve statistical significance. Finally, the likelihood ratio test (1.76 on 2 df, *p* = 0.40) showed an acceptable homogeneity among these three patient groups. Concordance was 0.533 (standard error = 0.037).

### Appendix A.4. Survival Analysis for the TKIBLI Regimen (Two Trials)

Finally, the third group of similar/identical treatments regarded blinatumomab combined with a second or third-generation TKI. In this group, we included two trials, namely blinatumomab combined with second-generation dasatinib, as reported by Chiaretti et al. [25], and blinatumomab combined with the third generation ponatinib, as reported by Jabbour et al. [26]. Quite interestingly, in this case, the two curves were nearly identical (Figure A3). Since no events were reported in the dasatinib+blinatumomab trial by Chiaretti et al. [25], neither the HR nor the likelihood ratio test could be computed. Likewise, medians could not be computed from these two curves. However, concordance was high (concordance = 0.799, standard error = 0.025).
